# Changes of Subjective Symptoms and Tear Film Biomarkers following Femto-LASIK

**DOI:** 10.3390/ijms23147512

**Published:** 2022-07-06

**Authors:** Teresa Tsai, Mohannad Alwees, Anika Rost, Janine Theile, H. Burkhard Dick, Stephanie C. Joachim, Suphi Taneri

**Affiliations:** 1 Experimental Eye Research Institute, University Eye Hospital, Ruhr-University Bochum, In der Schornau 23-25, 44892 Bochum, Germany; teresa.tsai@rub.de (T.T.); mohannad.alwees@kk-bochum.de (M.A.); janine.theile@rub.de (J.T.); burkhard.dick@kk-bochum.de (H.B.D.); 2 Center for Refractive Surgery Münster, Eye Department, St. Francis-Hospital Münster, Hohenzollernring 70, 48145 Münster, Germany; rost@refraktives-zentrum.de

**Keywords:** CGRP, discomfort, dry eye, Femto-LASIK, OSDI, refractive surgery, Schirmer test, tear film

## Abstract

Femtosecond laser-assisted in situ keratomileusis (Femto-LASIK) represents a common treatment modality in refractive surgery and shows excellent results in terms of safety, efficacy, predictability, and long-term stability. However, patients may be affected by dry eye symptoms. The aim of this study was to identify a potential association between subjective dry eye symptoms, objective dry eye markers, and possible changes in the tear film, which could be a target for future therapy development. Therefore, clinical (dry eye) examinations (OSDI, Schirmer test, lissamine green and fluorescein staining, BUT, visual acuity) were carried out before LASIK as well as 5 and 90 days post-OP. The dry eye marker MMP-9, cytokines (IL-1β, IL-8), and pain markers (NGF, CGRP) were quantified in tear samples with immunoassays. In addition, correlation analyses were performed. Clinical examinations revealed an upregulated OSDI score 5 days post-OP and an increased lissamine green staining score 90 days post-OP. Downregulated CGRP levels were noted 5 days post-OP, while other protein markers were not significantly altered after Femto-LASIK. Hence, Femto-LASIK surgery induced subjective symptoms like that of dry eye which could objectively rather be classified as Femto-LASIK-related discomfort. In the future, this could possibly be better detected and treated using pain markers such as CGRP.

## 1. Introduction

Refractive errors are a leading cause of reversible visual impairment around the globe and are a common problem in all age groups. In Europeans between 25 and 90 years of age, about 31% are myopic and 25% are hyperopic [1]. In 2011, approximately 69% of individuals above the age of 16 years used glasses or contact lenses in Germany [2].

There are plenty of options to correct refractive errors, the most common ones are glasses and contact lenses, however, corneal- or lens-based refractive surgery are established options. Although glasses provide a safe and effective method to correct refraction errors, many patients look for other methods, because of difficulties when using glasses during work, sports, or due to personal as well as social aspects. Contact lenses are an alternative, but also have some disadvantages. They can be care and time demanding as well as not being manageable for everyone. Moreover, they can cause ocular complications. Discomfort, dry eye symptoms, corneal infiltrates, and giant papillary conjunctivitis are among the most common complications of contact lenses. In addition, serious complications, such as corneal neovascularization, corneal abrasion, and infectious keratitis may also occur [3]. A surgical solution is therefore an attractive option for many patients.

The last century witnessed the development of refractive surgery, starting from manual methods such as refractive corneal sculpting and arcuate incisions [4]. However, the revolutionary progress was the use of laser as a tool to reshape the cornea. The 193 nm argon excimer laser can precisely sculpt the stroma and was first used for photorefractive keratectomy (PRK). In PRK, the surgeon removes the epithelium, and the laser ablates the stroma to re-shape the anterior corneal surface. This procedure was further refined, by creating a superficial flap in the cornea with a microkeratome, which is opened before applying the excimer laser on the stromal bed. The flap is reflected at the end of the procedure and heals without sutures. This treatment modality is called laser-assisted in situ keratomileusis (LASIK). LASIK has many advantages over PRK. The healing of the corneal epithelium and the improvement of vision are significantly faster. In addition, LASIK is more comfortable for the patient during the early post-operative period and complications are rare [5]. A further milestone in refractive surgery was the introduction of the femtosecond laser, which uses ultra-short laser pulses to precisely separate tissues. The femtosecond laser technology allowed the creation of LASIK flaps of a uniform thickness. Hence, Femto-LASIK is an effective and safe refractive surgical modality. However, some limitations still exist, such as post-operative inflammation and the development of dry eye symptoms. These dry eye symptoms are often perceived right after surgery and usually last about a month or longer. Nonetheless, some patients experience more severe symptoms than others and/or for a longer period of time, negatively affecting their quality of life [6,7]. A better understanding of the healing process and identification of biomarkers that might detect patients at risk of developing severe postoperative symptoms in advance may aid in patient identification as well as pre- and postoperative therapy of those symptoms.

At present, there may be a discrepancy of objective signs and patients reported subjective symptoms of dry eyes, especially after Femto-LASIK. A classic dry eye examination includes tests such as the tear film break-up time (BUT), Schirmer test (tear film secretion rate), tear film osmolarity, fluorescein as well as lissamine green staining, and meibomian gland secretion scoring [8]. However, most tests lack objectivity and specificity or are prone to user-dependent analytical errors [9]. In addition, there is often a lack of correlation between objective signs (test results) and subjective symptoms as evaluated using the ocular surface disease index (OSDI) questionnaire.

Tear-based biomarkers herald a new era in diagnostics. Tear fluid is easily accessible, reflecting pathophysiological changes of eye diseases. In particular, Schirmer test strips, which are normally discarded after measuring tear secretion, can be used to obtain analytical samples. Protein concentrations can change in the natural course of diseases or because of surgery. In recent years, several dry eye biomarkers were identified in tear fluid. Tear-derived inflammatory biomarkers include tumor necrosis factor alpha (TNF-α), interleukin 6 (IL-6), IL-8, and IL-17, which are all elevated in dry eyes [9,10,11]. In addition, matrix metalloproteinase-9 (MMP-9), a gelatinase produced in the corneal epithelium and activated in the tear film, is currently used as a marker for the diagnosis of dry eye [12,13]. Since corneal neuralgia has been reported in some patients after refractive surgery, this adverse event is currently under investigation and the nerve growth factor (NGF) can be an indicator of corneal nerve damage [14]. In addition to NGF, the calcitonin gene-related peptide (CGRP) is also an interesting starting point, as it appears to be involved in corneal nociception and dry eye symptoms [15].

Dry eyes after refractive surgery have already been evaluated extensively using the classic examination methods [16,17], however, with contradictory conclusions [18,19]. Therefore, a more accurate and, above all, objective assessment is required. In addition, a detailed analysis of pain markers after refractive laser surgery is still lacking. For this reason, in the current study, both tear film samples and classical dry eye examinations were recorded and compared over time after Femto-LASIK. These investigations should contribute to an improved and comprehensive understanding of the post-operative (post-OP) patient condition.

## 2. Results

### 2.1. Clinical Patient Data

In total, fourteen patients, ten male and four female, were included in this study. Examinations were carried out preoperatively (pre-OP) as well as in a follow-up period of 5 days (5 days post-OP) and 90 days (90 days post-OP) after surgery. The patient cohort had an average age of 37.26 ± 11.94 years and a median age of 34. Data from the right eye of all patients were included in the study (Table 1).

### 2.2. Classical Dry Eye Examinations after Femto-LASIK

Patients undergoing Femto-LASIK were examined before, 5 and 90 days after surgery using classic dry eye examinations (Table 2).

**Figure 1:**
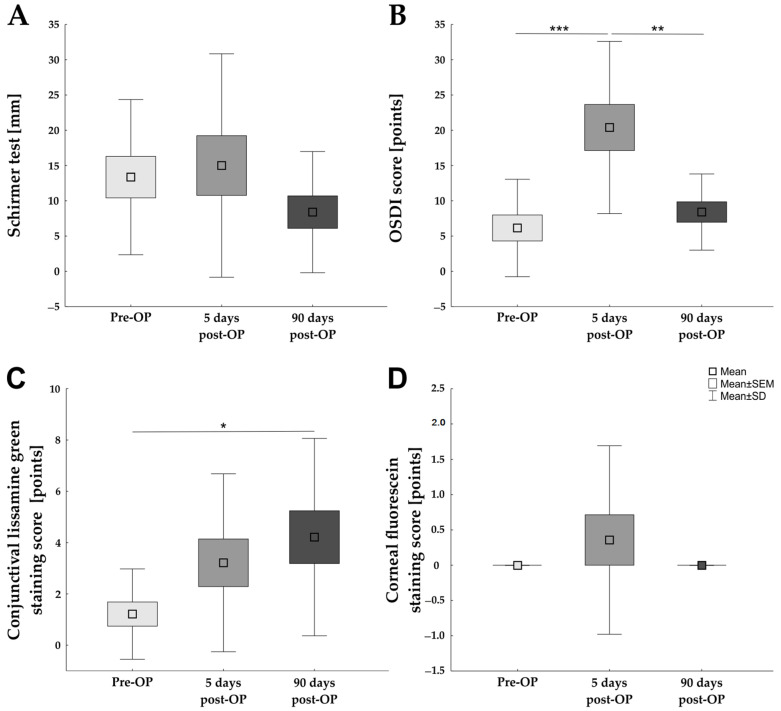
Classic dry eye examinations over time. (**A**) No significant difference in the Schirmer test values was detected between pre-OP, 5 days post-OP, and 90 days post-OP. (**B**) The OSDI score was significantly increased at 5 days post-OP in comparison to pre-OP and 90 days post-OP, respectively. (**C**) The conjunctival lissamine green staining showed an increasing trend postoperatively and was significantly upregulated 90 days post-OP in comparison to the preoperative investigations (**D**) Results of the corneal fluorescein staining score revealed no differences between the three investigation points in time. Values are mean ± SEM ± SD; * *p* < 0.05; ** *p* < 0.01; *** *p* < 0.001. Abbreviation: OSDI = ocular surface disease index.

Schirmer test values did not differ significantly between pre-OP (13.36 ± 11.00 mm) and 5 days post-OP (15.00 ± 15.84 mm; *p* = 0.932) and neither between pre-Op and 90 days post-OP (8.39 ± 8.59 mm; *p* = 0.533; Figure 1A).

In contrast, the OSDI score was significantly increased 5 days post-OP (20.39 ± 12.21 points) in comparison to the pre-OP score (6.15 ± 6.90 points; *p* < 0.001) and the 90 days post-OP score (8.41 ± 5.40 points; *p* = 0.002), respectively. No difference in the OSDI score was detectable between the pre-OP and 90 days post-OP investigation point (*p* = 0.770; Figure 1B). Based on the fact that the OSDI score showed significant alterations over time, the answers of the three OSDI subscales were investigated separately: vision-related function, ocular symptoms, and environmental triggers (Table 3). The results of the vision related functions of the OSDI revealed a significant increase in the score 5 days post-OP (27.86 ± 15.15 points) in comparison to the pre-OP situation (7.14 ± 8.02 points; *p* < 0.001) and the 90 days post-OP situation (11.07 ± 8.13 points; *p* < 0.001), respectively. In contrast, no significant differences could be detected between pre-OP and 90 days post-OP regarding the vision-related function (*p* = 0.613). Analyzing the ocular symptoms subscale, neither a difference between the score pre-OP (4.91 ± 9.23 points) and 5 days post-OP (16.96 ± 24.03; *p* = 0.140) nor between pre-OP and 90 days post-OP (8.33 ± 11.95 points; *p* = 0.846) was observed. In addition, the comparison between 5 days post-OP and 90 days post-OP detected no differences (*p* = 0.354). The examination of the environmental triggers revealed a similar picture, no differences could be observed between pre-OP (4.76 ± 15.58 points), 5 days post-OP (9.62 ± 27.55 points; *p* = 0.771), and 90 days post-OP (3.87 ± 5.77 points; *p* = 0.991).

When analyzing the conjunctiva via lissamine green staining, a significantly increased score 90 days post-OP (4.21 ± 3.85 points) in comparison to the pre-OP values (1.21 ± 1.76 points; *p* = 0.042) was revealed. Lissamine green staining at 5 days post-OP (3.21 ± 3.47 points), was in between pre-OP (*p* = 0.227) and 90 days post-OP (*p* = 0.682; Figure 1C) without a statistically significant difference.

Examination of the cornea using fluorescein staining did not identify any differences between the scores at pre-OP (0.00 ± 0.00 points) and 5 days post-OP (0.36 ± 1.34 points; *p* = 0.446) nor between pre-OP and 90 days post-OP (0.00 ± 0.00 points; *p* = 1.000). Furthermore, no alterations could be recorded between the two points in time after Femto-LASIK (*p* = 0.446; Figure 1D).

The stability of the tear film was analyzed via BUT test. No significant difference in the break-up time was observed between pre-OP (9.20 ± 3.77 sec) and 5 days post-OP (8.75 ± 3.55 sec; *p* = 0.954) nor 90 days post-OP (7.00 ± 3.55 sec; *p* = 0.317). The comparison of the two BUT values after Femto-LASIK revealed no significant difference either (*p* = 0.443; Table 2).

Visual acuity values were also determined with best spectacle correction at distance preoperatively and uncorrected postoperatively as this is what the patient perceives subjectively although this is methodically not identical. There was a significant reduction in visual acuity comparing corrected vision pre-OP (1.41 ± 0.21 decimal scale) and uncorrected vision 5 days post-OP (1.07 ± 0.30 decimal scale; *p* = 0.030); however, not between pre-OP and 90 days post-OP (1.33 ± 0.39 decimal scale; *p* = 0.825). Both post-OP visual acuities were comparable (*p* = 0.109; Table 2).

### 2.3. Protein Alterations after Femto-LASIK

**Figure 2:**
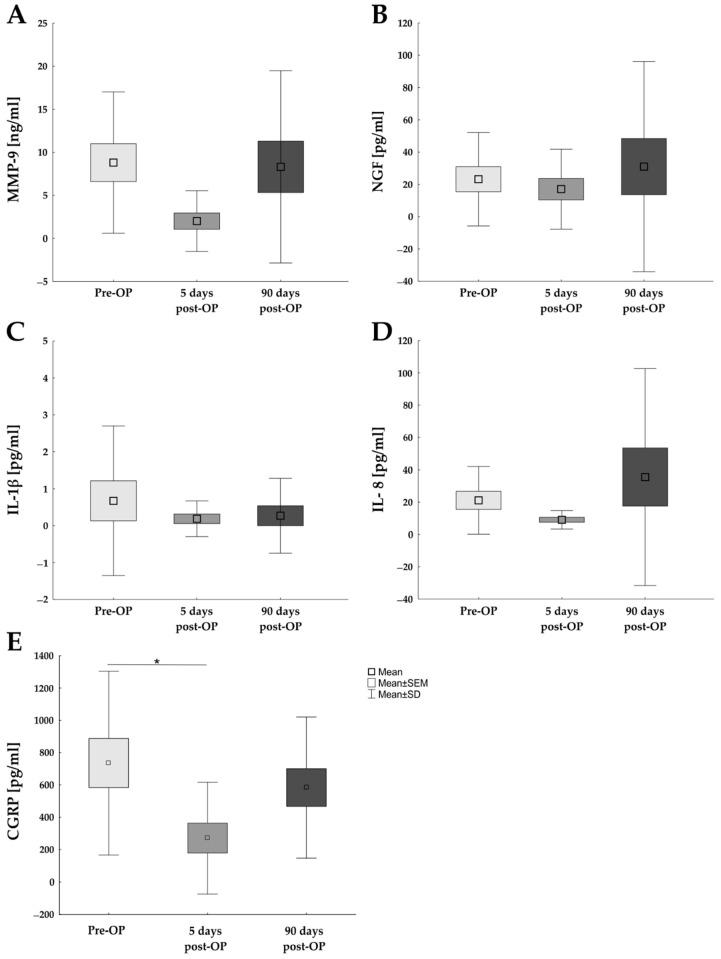
Protein expression in tear film before and after Femto-LASIK. (**A**) Evaluation of MMP-9 values pre-OP as well as 5 and 90 days after Femto-LASIK revealed no significant differences. (**B**) The NGF level at all three investigation points was nearly the same. (**C**) The amount of the pro-inflammatory cytokine IL1-β was not altered following Femto-LASIK. (**D**) The same levels for the pro-inflammatory chemokine IL-8 were identified in all samples. (**E**) The CGRP level at 5 days post-OP was significantly downregulated in comparison to pre-OP, but not in comparison to 90 days post-OP. Values are mean ± SEM ± SD; * *p* < 0.05. Abbreviations: CGRP = calcitonin gene-related peptide; IL = interleukin; MMP-9 = matrix metalloproteinase-9; NGF = nerve growth factor.

Tear fluid samples were eluted from Schirmer stripes before, as well as 5 and 90 days after Femto-LASIK surgery and were analyzed via enzyme-linked immunosorbent assay (ELISA) measurements (Figure 2 and Table 4).

Examining the level of MMP-9, a common dry eye marker [12], pre-OP (8.81 ± 8.20 pg/mL), 5 days post-OP (2.01 ± 3.53; *p* = 0.088), and 90 days post-OP (8.32 ± 11.17; *p* = 0.986), revealed no significant differences between these examination points (Figure 2A).

The level of NGF, a key regulator of immune reaction, trophic support, healing of ocular surface, corneal sensitivity, and tear film function [20], was analyzed in all tear fluid samples. The level of NGF 5 days post-OP (23.21 ± 28.95 pg/mL) was nearly the same as pre-OP (23.21 ± 28.95 pg/mL; *p* = 0.927). In addition, 90 days post-OP, the NGF level (31.05 ± 65.08 pg/mL) was unaltered in comparison to pre-OP (*p* = 0.883) and 5 days post-OP (*p* = 0.676; Figure 2B).

Inflammation with cytokine release may be an underlying cause of dry eye disease [10]. Therefore, levels of the inflammatory cytokines IL-1β and IL-8 were analyzed in the tear film samples at all three points in time. The pre-OP samples revealed the highest IL-1β level (0.67 ± 2.03 pg/mL). Nevertheless, a significant difference to the 5 days post-OP samples (0.19 ± 0.48 pg/mL; *p* = 0.717) and 90 days post-OP samples (0.27 ± 1.01 pg/mL; *p* = 0.707) was not detected (Figure 2C).

Regarding the chemokine IL-8 quantity in the tear fluid, no significant differences between the examinations could be recorded (*p* > 0.100). Similar levels of IL-8 could be detected in the samples pre-OP (21.13 ± 20.99 pg/mL) as well as 5 days post-OP (9.11 ± 5.72 pg/mL) and 90 days post-OP (35.53 ± 67.11 pg/mL; Figure 2D).

In addition, CGRP was investigated in tear samples. Interestingly, the CGRP level 5 days post-OP (271.51 ± 344.73 pg/mL) was significantly downregulated in comparison to pre-OP (735.39 ± 568.60 pg/mL; *p* < 0.029), but not in comparison to 90 days post-OP situation (583.74 ± 436.14 pg/mL; *p* = 0.183). Comparing the CGRP values pre-OP and 90 days post-OP, no differences were detected (*p* = 0.666; Figure 2E).

### 2.4. Correlation Analysis between OSDI Score and Objective Examinations and Protein Alterations over Time

The correlation analysis between the OSDI score and the Schirmer test, conjunctival lissamine green staining, corneal fluorescein staining, BUT, and visual acuity revealed no correlation preoperatively. In addition, 5 days post-OP, when the OSDI score was significantly higher in comparison to the other points in time, no correlation to the objective examinations was detected. However, 90 days post-OP, there was a trend towards a negative correlation between the OSDI score and the BUT (*p* = 0.055). A correlation between the OSDI score and the other examination values could not be observed at this time (Table 5).

Furthermore, correlation analyzes were carried out between the OSDI score and the examined tear film protein concentrations. No correlation between OSDI score and measured protein concentrations could be identified at any of the three time points. Only the IL-8 concentration 90 days post-OP showed a tendency towards a negative correlation with the OSDI score (*p* = 0.081; Table 5).

### 2.5. Correlation Analysis between Lissamine Green Staining and Protein Levels

Since the lissamine green staining at 90 days post-OP was significantly elevated compared to pre-OP, a correlation analysis between lissamine green staining and protein levels was performed. However, no correlation between the lissamine green staining and the tear film protein levels could be detected at any of the three time points examined (Table 6).

## 3. Discussion

Refractive surgery provides patients with refractive errors a corrective solution beyond glasses or contact lenses. Hence, it is crucial to optimize the results of this elective surgery and avoid complications and unwanted side effects. One of the most common side effects that may cause discomfort for patients are dry eye symptoms. At present, classic objective examinations of dry eye signs are routinely used to record the subjective sensation of dry eyes in patients after Femto-LASIK. However, these tests often lack a correlation between objective signs and subjective symptoms. Therefore, we separately studied the subjective symptoms and objective signs of dry eye before and after surgery. In parallel, we analyzed selected proteins in the patients’ tears. The aim was to obtain a more comprehensive overview of the condition before and after the Femto-LASIK, searching for a possible connection between these parameters.

The OSDI questionnaire is a common tool in the clinic as well as clinical trails and is regarded as valid and reliable to evaluate the severity of dry eye disease [21]. Analyzing the total OSDI score, we found a higher score 5 days after surgery in comparison to the values before surgery. The total OSDI score returned to baseline after 90 days. A study by Elmohamady et al., which evaluated femtosecond laser in flap and cap creation in corneal refractive surgery for myopia, was also able to show a statistically significant increase in the OSDI score one month postoperatively compared to the preoperative values. However, in this study, the mean OSDI score returned to preoperative levels later, after 6 months [22]. Comparing the three subclasses of the OSDI score, only the vision-related score was elevated 5 days post-OP, whereas the subclasses symptoms and environmental triggers were not altered between the three examination points. The International Task Force Guidelines for severity of dry eye symptoms and effects on vision-related functioning in the United States, however, showed that with respect to the classic dry eye disease, all three parameters worsened with increasing severity level [23]. Interestingly, a study by Gong et al., which evaluated dry eye after refractive surgery according to the meibomian gland status, also found an upregulation of the total OSDI score one month after surgery, which gradually returned to baseline level after three months. Moreover, they also detected significant higher vision-related scores in the group with the highest meibomian gland dysfunction after one month, whereas the environmental trigger score was upregulated in this group only after three months [24]. Therefore, the results of our and this study show that symptoms after Femto-LASIK surgery are not identical to those of classic dry eye disease and may require a different treatment due to their different etiology.

In addition to the OSDI questionnaire, we performed numerous classic dry eye examinations, but the objective clinical measurements showed no significant difference, with one exception. The Schirmer test did not show a significant alteration between pre-OP and both post-OP measurements. The fluorescein staining of the cornea was seldomly detectable in our study. The BUT test was also comparable in all analyzed examination points. Only the lissamine staining of the conjunctiva showed an increase in the post-OP examinations, 90 days after surgery. Therefore, our classical objective measurements do not correspond well to the subjective symptoms detected by the OSDI questionnaire. This is in line with several other previous studies which also have shown that an association between dry eye symptoms, as recorded by OSDI, and clinical measurements are often low and inconsistent [25,26].

In the next step, we analyzed protein changes in the tear film over time. Surprisingly, despite the changed OSDI score, we could not identify any change of the classical dry eye marker MMP-9 [12]. This result contrasts with a study by González-Pérez et al., which reported an increase in the MMP-9 concentration months after LASIK surgery. It needs to be pointed out that their patients were myopic, the surgeons used a microkeratome and not a femto-laser to make the flap, and a micropipette was used to collect the tear fluid [27]. Thus, there are several differences to our study. However, a study following femtosecond laser-assisted cataract surgery showed results similar to ours, eye discomfort was observed without an increase in MMP-9 levels. The authors concluded that ocular discomfort experienced in patients post-surgery may be due to an etiology other than the development or worsening of dry eye disease [28]. Thus, while MMP-9 is considered as a classic dry eye marker and clinical tests are commercially available, its usefulness as a biomarker after refractive surgery is difficult to evaluate due to a wide variability in levels in some patients. It is therefore controversially discussed in the research community [28,29,30].

In addition to MMP-9, we also analyzed the level of the cytokines IL-1β and IL-8 after Femto-LASIK, which are also known to be elevated in dry eye [11,31]. These ELISA measurements also did not detect any regulations between the examined points in time. One reason for the lack of cytokine alterations could be the point of investigation. Resan et al. were able to show that the acute phase of wound-healing after excimer laser treatment (1–24 h) is accompanied by an increased inflammatory response (IL-1β, IL-8, TNF-α) [32]. In addition, our routine local application of anti-inflammatory and antibiotic eye drops in the first ten days after surgery may have prevented severe inflammatory events. However, it can be stated that alterations in IL-1β and IL-8 levels are not the reason for the impairments detected by the OSDI score in our study.

Furthermore, we investigated the level of two pain markers in the tear fluid. NGF has been shown to be upregulated in conditions that damage corneal nerves. In these cases, NGF stimulates corneal sensitivity and nerve regeneration [14,33]. Analyzing the NGF level in tear fluid, we did not reveal any differences between pre-OP and both postoperative points. This result contrasts with two studies which observed an upregulation in the NGF level after the treatment of myopia via Femto-LASIK [34,35]. But our study analyzed hyperopia patients. Interestingly, the increase in NGF levels in both aforementioned studies was accompanied by lower BUT and Schirmer values, which we also did not observe. Another study, however, using small incision lenticule extraction for the treatment of myopia, also found no changes in tear film NGF levels neither at one week nor at three months after surgical treatment [36].

Moreover, we analyzed CGRP levels in the tear fluid. CGRP is a constitutively expressed neuropeptide that is secreted in tears to maintain the integrity of the corneal epithelium [37]. Using ELISA measurements, we were able to demonstrate a reduction in the CGRP level 5 days post-OP. After 90 days, the CGRP level almost reached the initial value. Analysis of the CGRP level after refractive surgery has so far been limited. A single-visit cross-sectional study investigating patients 12 months after Femto-LASIK and healthy participants detected an upregulated CGRP level in the Femto-LASIK cohort [38]. Moreover, in patients with myopia and treated with SMILE, the CGRP level remained stable after surgery [36]. Interestingly, in tear samples of patients with severe dry eye disease (Sjögren’s syndrome) a decreased CGRP level could be revealed via ELISA measurements. The authors concluded the CGRP could be related to impaired lacrimal function and a potential marker of dry eye severity [39].

In summary, correlation analyzes between OSDI score and classical examinations as well as ELISA measurements in our study were not able to reveal a connection between the subjective symptoms on the one hand and the analyzed objective markers on the other hand. This indicates a different etiology of similar symptoms of dry eye disease and status post Femto-LASIK.

Our study had some limitations, including a relatively small sample size and only two postoperative time points. In addition, we used anti-inflammatory corticosteroid and antibiotic eye drops in the first ten days after the procedure as well as hyaluronic acid for a longer period as is the established standard of care worldwide. The influence of this medication on our study results remains elusive as it could have caused discomfort (by a different acidity than the ocular surface) and interfered with osmolarity, viscosity and cohesivity of the tear film. In addition, this corticosteroid treatment could also be the reason behind the absence of classic dry eye signs, such as MMP-9 alteration and inflammatory cytokine elevations. In line, studies in experimental dry eye mice found that corticosteroid and doxycycline suppress MMP-9 and inflammatory cytokines expression [40]. 

Based on the available data, however, it appears that the Femto-LASIK surgery does not lead to classical dry eye symptoms, but rather causes discomfort which may be better detected and treated using pain markers. For this purpose, further analyzes in the future of the CGRP level as well as other potential neuromodulators such as substance *p* should be investigated, ideally at more points in time.

## 4. Materials and Methods

### 4.1. Subjects, Clinical Examinations, and Sample Collection

Approval from the ethic committee of the Medical Association of Westphalia-Lippe (Münster, Germany) was obtained for this study (approval number: 2019-489-f-S). The study was registered in the German Register of Clinical Studies (DRKS): DRKS00022137. Written informed consent was obtained from all patients prior to the study. In addition, the tenets of the Declaration of Helsinki were observed.

All eyes were hyperopic with or without astigmatism and underwent routine Femto-LASIK surgery at the Eye Center at the St. Franziskus Hospital, Münster. The uniformity of the treated ametropia allowed the examination of the connection between dry eye symptoms, tear film marker and the Femto-LASIK surgical technique without being influenced by the form of ametropia. We excluded patients with systemic or corneal diseases that are contraindications for Femto-LASIK. Postoperatively, all eyes were routinely treated over 10 days with unpreserved ofloxacin 4 times a day and unpreserved dexamethasone 4 time a day. Artificial tears based on hyaluronic acid were recommended to be applied hourly for 10 days and 5 times a day thereafter. The study included 14 patients.

**Figure 3:**
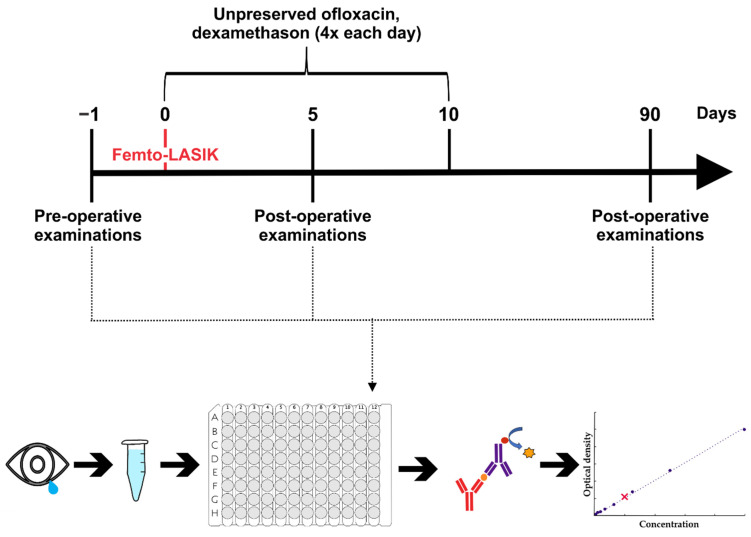
Experimental setup for evaluation of dry eye symptoms and protein alterations after Femto-LASIK. Patients were examined both preoperatively as well as 5 and 90 days after LASIK. Both, classic dry eye examinations and analyzes of the protein concentration in the tear film were carried out. Tear film was obtained using Schirmer strips, eluted and transferred to an ELISA plate. The quantity of altered proteins was determined using specific antibodies in commercially available ELISA kits.

Prior to sample collection, clinical data including age, gender, eye, known chronic diseases and regular medication were collected for each patient, and clinical examinations were carried out. The clinical examination included the Schirmer test, the Ocular Surface Disease Index questionnaire (OSDI), and an examination of the tear film and cornea with fluorescein eye drops, in which we measured the tear break up time (BUT) and documented the staining of the cornea. Moreover, the conjunctivae were analyzed using lissamine green staining. Moreover, visual acuity was documented. The clinical examinations were repeated 5 and 90 days after surgery (Figure 3).

The Schirmer test strips were also used to collect the tear samples. These strips were transferred to the research laboratory of the eye clinic at the Ruhr-University Bochum, where the tear fluid was eluted, frozen, and stored at −80 °C until analysis by ELISA measurements were performed (Figure 3).

### 4.2. Measurement of Cytokines in Vitreous Samples

All tear film proteins were analyzed and quantified using commercially available ELISA kits (Table 7). Each assay was performed according to the manufacturer’s instructions. For CGRP measurements, samples were diluted (Table 7) using a sample diluent buffer just prior to the assay. For all other measurements, undiluted samples were used. All evaluations were performed on a microplate reader (AESKU Reader with Gen5 ELISA Software, AESKU. DIAGNOSTICS, Wendelsheim, Germany).

### 4.3. Statistical Analysis

All statistical analyses were performed using the commercial predictive analytic Statistica program (Version 14; Dell, Tulsa, OK). Significant differences in protein and cytokine concentration between the points in time were compared by ANOVA followed by Tukey post-hoc test. *p*-values below 0.05 were considered to be statistically significant: * *p* < 0.05, ** *p* < 0.01, *** *p* < 0.001. Graphs display data mean ± standard error mean (SEM ± standard deviation (SD).

Pearson’s correlation coefficient (r) was calculated for the analyses of the associations between the patient’s individual OSDI score, protein level or classical examinations. Pearson’s correlation coefficient was also calculated between lissamine green staining score and protein levels.

## 5. Conclusions

In our study, subjective symptoms similar to those of dry eye could be detected via OSDI questionnaire, but alterations in objective clinical dry eye signs were not observed. Thus, subjective symptoms detected with the OSDI score are not specific for dry eyes, especially not after Femto-LASIK surgery, since the symptoms of tear film deficiency overlap with those of irritation caused by surgical wound healing (as observed also after cataract surgery). We assume, it would be more appropriate to avoid the term “LASIK-induced dry eye” and use “LASIK-induced discomfort” instead. Interestingly, examinations of tear film proteins only showed a significant downregulation of the pain marker CGRP after Femto-LASIK. The administration of anti-inflammatory and antibiotic drugs (given for 10 days) could be an explanation for the absence of inflammatory markers as well as the unaltered MMP-9 levels. The regulation of CGRP indicates that pain markers may represent a new way to identify and treat post-Femto-LASIK discomfort.

## Figures and Tables

**Table 1:** Clinical patient data for all groups. Abbreviations: M = male, F = female; OD = right eye; OS = left eye.

Samples (pre-OP/5 post-OP/90 post-OP)	14
Mean age (year) Median age (year)	37.26 ± 11.94 34
Gender (M/F)	10/4
Eye (OD/OS)	14/0

**Table 2:** Classical dry eye examination (mean ± SD) before as well as 5 and 90 days after Femto-LASIK. Visual acuity pre-OP was best corrected distance vision and uncorrected vision post-OP. Significant *p*-values are in bold. Abbreviations: BUT = tear break-up time; OSDI = ocular surface disease index.

	Point of Analysis			*p*-Value		
	Pre-OP	5 Days Post-OP	90 Days Post-OP	Pre-OP vs. 5 Days Post-OP	Pre-OP vs. 90 Days Post-OP	5 Days Post-OP vs. 90 Days Post-OP
Number of samples	14	14	14			
Conjunctival lissamine green staining [points]	1.21 ± 1.76	3.21 ± 3.47	4.21 ± 3.85	0.227	**0.042**	0.682
Corneal fluorescein staining [points]	0 ± 0	0.36 ± 1.34	0 ± 0	0.446	1.000	0.446
BUT [sec]	9.20 ± 3.77 (n = 10)	8.75 ± 3.55 (n = 12)	7.00 ± 3.55 (n = 14)	0.954	0.317	0.443
Schirmer test [mm]	13.36 ± 11.00	15.00 ± 15.84	8.39 ± 8.59	0.932	0.533	0.334
OSDI score [points]	6.15 ± 6.90	20.39 ± 12.21	8.41 ± 5.40	**<0.001**	0.770	**0.002**
Visual acuity [decimal scale]	1.41 ± 0.21	1.07 ± 0.30	1.33 ± 0.39	**0.030**	0.825	0.109

**Table 3:** Detailed OSDI score (mean ± SD) before as well as 5 and 90 days after Femto-LASIK. Significant *p*-values are in bold. Abbreviation: OSDI = ocular surface disease index.

	Point of Analysis			*p*-Value		
	Pre-OP	5 Days Post-OP	90 Days Post-OP	Pre-OP vs. 5 Days Post-OP	Pre-OP vs. 90 Days Post-OP	5 Days Post-OP vs. 90 Days Post-OP
OSDI total score [points]	6.15 ± 6.90	20.39 ± 12.21	8.41 ± 5.40	**<0.001**	0.770	**0.002**
OSDI: vision-related function [points]	7.14 ± 8.02	27.86 ± 15.15	11.07 ± 8.13	**<0.001**	0.613	**<0.001**
OSDI: ocular symptoms [points]	4.91 ± 9.23	16.96 ± 24.03	8.33 ± 11.95	0.140	0.846	0.354
OSDI: environmental triggers [points]	4.76 ± 15.58	9.62 ± 27.55	3.87 ± 5.77	0.771	0.991	0.696

**Table 4:** Protein concentration in tear film samples before as well as 5 and 90 days after Femto-LASIK, measured via ELISA (mean ± SD). Significant *p*-values are in bold. Abbreviations: CGRP = calcitonin gene-related peptide; IL = interleukin; MMP-9 = matrix metalloproteinase-9; NGF = nerve growth factor.

	Cytokine Concentration			*p*-Value		
Cytokine	Pre-OP	5 Days Post-OP	90 Days Post-OP	Pre-OP vs. 5 Days Post-OP	Pre-OP vs. 90 Days Post-OP	5 Days Post-OP vs. 90 Days Post-OP
CGRP [pg/mL]	735.39 ± 568.60	271.51 ± 344.73	583.74 ± 436.14	**0.029**	0.660	0.183
IL-1β [pg/mL]	0.67 ± 2.03	0.19 ± 0.48	0.27 ± 1.01	0.606	0.707	0.985
IL-8 [pg/mL]	21.13 ± 20.99	9.11 ± 5.72	35.53 ± 67.11	0.717	0.622	0.212
MMP-9 [pg/mL]	8.81 ± 8.20	2.01 ± 3.53	8.32 ± 11.17	0.088	0.986	0.121
NGF [pg/mL]	23.21 ± 28.95	17.09 ± 24.76	31.05 ± 65.08	0.927	0.883	0.676

**Table 5:** Correlation between individual clinical measurements or protein concentration and ocular surface disease index (OSDI) score. Abbreviations: BUT = tear break-up time; CGRP = calcitonin gene-related peptide; IL = interleukin; MMP-9 = matrix metalloproteinase-9; NGF = nerve growth factor.

	OSDI Score								
	Pre-OP			5 Days Post-OP			90 Days Post-OP		
	r	*p*	r^2^	r	*p*	r^2^	r	*p*	r^2^
BUT	0.490	0.151	0.240	0.286	0.368	0.082	−0.524	0.055	0.275
Conjunctival lissamine green staining	−0.141	0.630	0.020	−0.430	0.125	0.185	0.291	0.312	0.085
Corneal fluorescein staining	0.000	0.000	0.000	0.344	0.228	0.119	0.000	0.000	0.000
Schirmer test	0.112	0.704	0.012	−0.021	0.944	0.000	0.169	0.563	0.029
Visual acuity	0.021	0.943	0.000	0.367	0.197	0.135	0.012	0.968	0.000
CGRP	−0.409	0.146	0.167	−0.139	0.637	0.019	0.197	0.520	0.039
IL-1β	0.441	0.115	0.194	0.042	0.888	0.002	0.106	0.718	0.011
IL-8	−0.164	0.575	0.027	−0.212	0.466	0.045	−0.482	0.081	0.233
MMP-9	−0.419	0.136	0.176	−0.272	0.347	0.074	−0.293	0.310	0.086
NGF	0.157	0.592	0.025	−0.102	0.728	0.011	0.413	0.142	0.171

**Table 6:** Correlation between protein concentration and lissamine green staining score. Abbreviations: CGRP = calcitonin gene-related peptide; IL = interleukin; MMP-9 = matrix metalloproteinase-9; NGF = nerve growth factor.

	Lissamine Green Staining								
	Pre-OP			5 Days Post-OP			90 Days Post-OP		
	r	*p*	r^2^	r	*p*	r^2^	r	*p*	r^2^
CGRP	0.246	0.396	0.061	−0.171	0.558	0.029	0.440	0.132	0.194
IL-1β	−0.216	0.458	0.047	−0.319	0.266	0.102	0.283	0.326	0.080
IL-8	0.248	0.393	0.061	−0.156	0.595	0.024	−0.356	0.212	0.127
MMP-9	0.116	0.693	0.013	0.050	0.865	0.002	−0.317	0.269	0.101
NGF	−0.180	0.537	0.033	−0.190	0.515	0.036	−0.272	0.347	0.074

**Table 7:** Applied ELISA assays including company, catalogue number, dilution, and references. Abbreviations: CGRP = calcitonin gene-related peptide; IL = interleukin; MMP-9 = matrix metalloproteinase-9; NGF = nerve growth factor.

**Protein**	**Company**	**Catalogue Number**	**Dilution**	**Reference**
β-NGF	Abcam	ab193760	undiluted	[41]
CGRP	Abcam	ab282299	1:2	n.a.
IL-1β/IL-1F2	R&D Systems	DLB50	undiluted	[42]
IL-8	Abcam	ab214030	undiluted	[43]
MMP-9	R&D systems	DMP900	undiluted	[44]

## Data Availability

The datasets generated during and/or analyzed during the current study are available from the corresponding author on reasonable request.
